# Beyond N staging in colorectal cancer: Current approaches and future perspectives

**DOI:** 10.3389/fonc.2022.937114

**Published:** 2022-07-18

**Authors:** Gianluca Arrichiello, Mario Pirozzi, Bianca Arianna Facchini, Sergio Facchini, Fernando Paragliola, Valeria Nacca, Antonella Nicastro, Maria Anna Canciello, Adele Orlando, Marianna Caterino, Davide Ciardiello, Carminia Maria Della Corte, Morena Fasano, Stefania Napolitano, Teresa Troiani, Fortunato Ciardiello, Giulia Martini, Erika Martinelli

**Affiliations:** ^1^ Oncology Unit, Department of Precision Medicine, Università degli Studi della Campania “Luigi Vanvitelli”, Naples, Italy; ^2^ Oncology Unit, Casa Sollievo della Sofferenza Hospital, San Giovanni Rotondo, Italy

**Keywords:** colorectal cancer, tumor staging, lymph node metastases, adjuvant treatment, TNM

## Abstract

Traditionally, lymph node metastases (LNM) evaluation is essential to the staging of colon cancer patients according to the TNM (tumor–node–metastasis) system. However, in recent years evidence has accumulated regarding the role of emerging pathological features, which could significantly impact the prognosis of colorectal cancer patients. Lymph Node Ratio (LNR) and Log Odds of Positive Lymph Nodes (LODDS) have been shown to predict patients’ prognosis more accurately than traditional nodal staging and it has been suggested that their implementation in existing classification could help stratify further patients with overlapping TNM stage. Tumor deposits (TD) are currently factored within the N1c category of the TNM classification in the absence of lymph node metastases. However, studies have shown that presence of TDs can affect patients’ survival regardless of LNM. Moreover, evidence suggest that presence of TDs should not be evaluated as dichotomic but rather as a quantitative variable. Extranodal extension (ENE) has been shown to correlate with presence of other adverse prognostic features and to impact survival of colorectal cancer patients. In this review we will describe current staging systems and prognostic/predictive factors in colorectal cancer and elaborate on available evidence supporting the implementation of LNR/LODDS, TDs and ENE evaluation in existing classification to improve prognosis estimation and patient selection for adjuvant treatment.

## Introduction

Colorectal cancer (CRC) represents the third most common cancer and the second leading cause of cancer-related death in the overall population, with nearly 1,148,515 new diagnosis and 576,858 deaths in 2020. Data have shown a slight difference between the two sexes; indeed, it takes up the second place for incidence and the third for mortality in women and the third for both incidence and mortality in men (1).

Despite being considered for many years an age-related neoplasia, in recent times there appears to be a decline in CRC incidence in the population over 50-year-old, balanced by an increase of new diagnosis in individuals younger than 50 years (2).

The 5-year survival rate has considerably increased during the past decades, reaching 63% all stages combined in 2021. There are however considerable variations amongst the 5-year survival rate depending on the TNM stage of the disease at moment of the diagnosis: as a matter of fact, it amounts to 91% in the localized disease (stage I-II), 72% in the regional disease (stage III) and it dramatically drops to 14% in the advanced disease (stage IV) (3, 4).

Complete resection of the primary tumor and regional lymph nodes remains the most effective therapy for early colon cancer. Adequate surgery also allows for evaluation of the resection specimen which is considered an essential step to define prognostic factors and predict disease recurrence after surgery, thus informing clinicians on potential benefits of adjuvant treatment.

Optimal management currently relies on the tumor-node-metastasis (TNM) staging system proposed by the American Joint Committee on Cancer (AJCC) and International Union Against Cancer (UICC), which assesses primary tumor (T), lymph node metastasis (N), and distant metastasis (M). This classification has now reached its eight iteration (5). Lymph node metastases, in particular, are considered a significant factor for predicting disease-free survival (DFS) and overall survival (OS) in patients with colorectal cancer without distant metastasis (6).

Beyond the above-mentioned TNM staging, other risk factors have shown an impact on the prognosis, particularly in stage II: pT4; inadequate lymphadenectomy (<12 lymph nodes); vascular invasion; lymphatic invasion; perineural invasion; high grade tumor; high preoperative CEA levels; tumor presentation with obstruction (7, 8). Moreover, MSI-H/MMRd status represents a molecular marker that has demonstrated to be related to a better prognosis in localized CRC and designates a subgroup of patients with less expected response to 5-fluorouracil-based chemotherapy (9).

However, definition of further pathological features can help improve existing classifications, to better identify patients with localized disease and a higher risk of relapse and to guide more accurately the choice of optimal adjuvant treatment.

In this review we will explore how emerging pathological characteristics, aside from existing biomarkers, can impact patient prognosis and how their factoring can improve disease management, and guide adjuvant strategies in colorectal cancer patients.

We will focus on the role of lymph node ratio, tumor deposits, extracapsular node extension.

**Figure 1:**
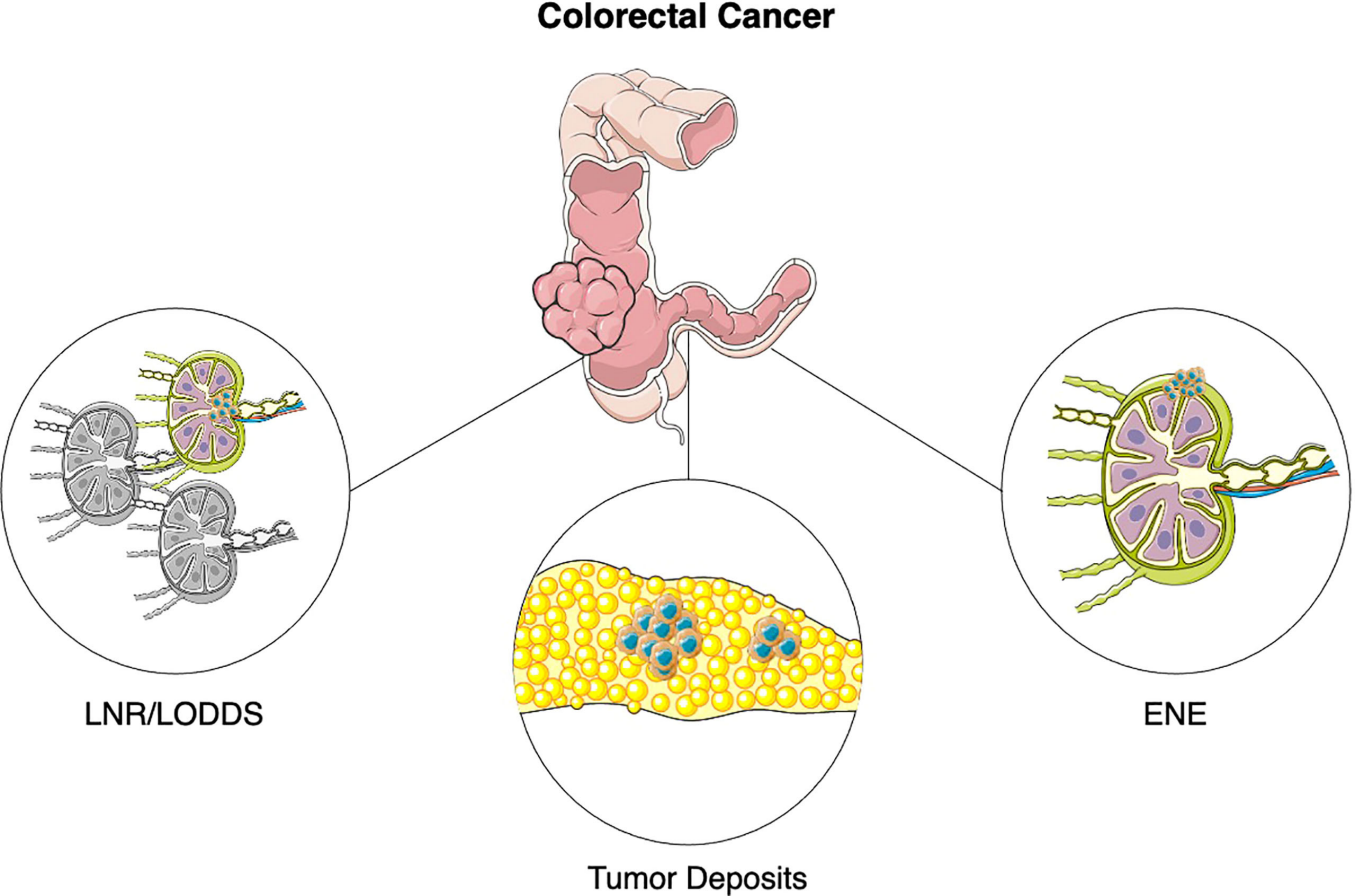
Emerging pathological features in colorectal cancer. Abbreviations: LNR, Lymph Node Ratio; LODDS, Logarithm of Positive Lymph Nodes; ENE, Extranodal extension.

A descriptive illustration is available in **Figure 1**.

### Methods

We reviewed the available literature on the use of lymph node ratio, tumor deposits and extracapsular node extension in CRC staging and overall management. We performed PubMed and Embase searches focused on these topics, selecting primary and review articles from peer-reviewed journals. Search terms included “lymph node ratio”, “log odds of positive lymph nodes”, “tumor deposits”, “extracapsular node extension”, “colorectal cancer”. We also searched PubMed and major oncology conferences for presentations pertinent to the matter of this review.

## Lymph node ratio and log odds of positive lymph nodes

### Lymph node ratio

Lymph Node Ratio (LNR) is defined as the ratio of metastatic lymph nodes (LN) over total LN examined (**Figure 1**). LNR has been established as a prognostic indicator in several non-colorectal malignancies, such as breast cancer, esophageal and gastric cancer, medullary and papillary thyroid cancer, non-small cell lung cancer, oropharyngeal cancer (10–16).

In colorectal cancer, even though pathological nodal stage remains one of the most important predictors of patient prognosis, several studies have tried to evaluate the potential of LNR as a prognostic marker.

When examining these results, it is important to underline that since 2002, three different American Joint Committee on Cancer (AJCC) staging classifications have been issued, with differences in stage grouping especially between 6^th^ and 7^th^ edition (17, 18).

All the studies revised are summarized in **Table 1**.

**Table 1:** Evidence regarding LNR/LODDS implementation.

Authors	Subject	Major Findings	Reference
Wang J et al.; Chin CC et al.; Ceelen W et al.	LNR	LNR is an independent and more accurate prognostic method for stage III colon cancer patients than AJCC TNM categories	(19–21)
Berger AC et al.	LNR	After curative resection for colorectal cancer, the LNR is an important prognostic factor and should be used to stratify patients receiving adjuvant treatment	(22)
In JP et al, Shimomura M et al., Li Destri G et al.	LNR	Adequate lymph node examination is important to ensure the prognostic value of LNR in patients with stage III colorectal cancer	(23–25)
Rosenberg R et al., Peschaud F et al.	LNR	LNR remains an independent prognostic predictor in colorectal cancer even when fewer than 12 LN are examined	(26, 27)
Isik A et al.; Parnaby CN et al.; Sabbagh C et al.; Shinto E et al.; Macedo F. et al; Zhang CH et al.	LNR	LNR impacts both DFS and OS in colon cancer patients; several cut-offs have been proposed to stratify patients	(28–33)
Sugimoto K et al; Wang LP et al.; Pei JP et al.; Yang LV et al.	LNR/LODDS	Implementation of LNR/LODDS improves prognostic accuracy of existing staging classifications	(34–37)
Madbouly KM et al.; Chen L et al.; Junginger et al.; Lykke J et al.; Karjol U et al.;	LNR	LNR can provide prognostic information in locally advanced rectal cancer and compensate for inadequate lymph node dissection in patients who did not receive preoperative therapy	(38–42)
Deng Y et al.; Alexandrescu ST et al.; Ahmad A et al.	LNR	High LNR correlates with burden of liver metastatic disease and predicts shorter RFS in patients undergoing curative resection	(43–45)
Mohan HM et al.; Jakob MO et al.	LNR	LNR is either equivalent or inferior to pathological nodal staging in patients with adequate LN harvesting	(46, 47)
Wang J et al; Fang HY et al.; Li T et al	LODDS	LODDS accurately predicts prognosis of patients with early-stage colon cancer	(19, 48, 49)
Occhionorelli S et al.	LODDS	LODDS is the only independent prognostic factor in patients with colon cancer receiving emergency surgery	(50)
Lee CW et al.; Xu T et al.	LODDS	LODDS accurately predicts prognosis of patients with locally advanced rectal cancer	(51, 52)
Baqar AR et al.; Song Y et al	LODDS/LNR	LODDS adds no prognostic information to LNR alone, which should be preferred due to ease of application	(53, 54)

DFS, Disease Free Survival; LN, Lymph Node; LNR, Lymph Node Ratio; LODDS, Logarithm of Positive Lymph Nodes; OS, Overall Survival.

Wang J et al. were among the first authors to show in 2008 the role of LNR as an independent predictor of survival in 24,477 stage III colon cancer patient from the SEER registry (19). Patients were stratified in 4 groups according to three different cutoffs (1/14, 0.25, 0.5) and LNR was deemed to be more accurate then TNM staging for stage IIIB and IIIC patients.

In 2005, Berger AC et al. published an analysis on stage II and III patients with colon cancer pooled from Intergroup trial 0089 of fluoropyrimidine-based adjuvant chemotherapy and proved LNR to be the most significant prognostic factor for both DFS and OS in patients with at least 10 LN sampled (22); interestingly, within the N1 and N2 classifications, dramatic changes were observed in recurrence rates based on the LNR value (less than 5%, 5% to 20%, 20% to 40%, or more than 40%). This work has the benefit of clarifying the prognostic relevance of LNR. Since all patients received adjuvant chemotherapy, it is unlikely that the impact of improved nodal staging is explained by more patients receiving intensified treatment. This means that a lower LNR, and consequent better prognosis, could be attributable to other variables, such as the quality of surgery performed.

It is a matter of debate whether the number of examined lymph nodes can influence the ability of LNR to stratify patients according to prognosis.

While in fact some authors suggested that discrimination provided by LNR is lost when less than 12 LN are examined (20, 23–25), work published by Rosenberg et al. showed that the LNR remained an independent predictor of outcome even when less than 12 nodes are examined and had better value than pathological nodal stage in the multivariate analysis (26).

The same observation was made by Peschaud F et al. in rectal cancer patients, where LNR predicted DFS and OS even when fewer than 12 LN were examined (27).

Ceelen W et al. eventually published in 2010 a systematic review based on 16 analyzed studies, including 33,984 patients with stage III colon or rectal cancer (21). In all the studies reviewed, LNR was an independent prognostic factor and allowed for a prognostic separation that was superior to that of the nodal stage alone in terms of OS, DFS and cancer specific survival.

Several trials have since been reported reinforcing the prognostic value of LNR in both early-stage colon and rectal cancer (28–30, 55). However, there is no consensus on the cut-off to use when applying LNR.

In 2014, a French regional study conducted by Sabbagh C et al. identified a 10% cutoff as optimum to distinguish between good and poor prognosis stage III colon cancer patients (31). This stratification allowed for significant correlation with 3-year OS and DFS.

Shinto E et al. proposed the use of different cut-offs to predict the prognosis of right or left-side primary colon cancer; by analyzing 5,463 patients with stage III colon cancer authors were able to stratify patients using values of 0.16 and 0.22 for right-sided and left-sided tumors, respectively (32).

Zhang CH et al. also designed a study to further validate the prognostic significance of LNR by evaluating 218,314 patients from the SEER database and 1,811 patients from three independent cohorts (33). Patients were divided into 5 groups according to LNR cutoffs previously investigated (0, 0.1-0.17, 0.18-0.41, 0.42-0.69, >0.7) and each group identified patients with worsening prognosis regardless of LN sampling.

Several attempts have been made to propose updates to pre-existing classifications by incorporating LNR information (34, 35).

Pei JP et al. developed a revised TLNR classification by combing tumor stage and LNR based on data from 62,294 early-stage colon cancer from the SEER registry and 3,327 additional patients from an external validation cohort (36). The novel classification was found to be superior to the AJCC 8^th^ TNM classification in predicting overall and disease-free survival.

Even though most efforts have focused on colon cancer, data has accumulated in rectal cancer patients as well (38–40).

For example, Junginger et al. demonstrated that LNR can provide prognostic information and thus compensate for inadequate lymph node dissection in patients with stage III rectal cancer who did not receive preoperative treatment (41).

Karjol U et al. recently published a systematic review and meta-analysis on this topic, encompassing 18 trials and 4,486 node-positive rectal cancer patients, confirming that a higher LNR was significantly correlated with worse OS and DFS (42).

However, not all the available evidence is in favor of LNR implementation in current staging systems. Mohan HM et al. suggested that LNR provides no additional information when compared with nodal staging, while Jakob MO et al. determined LNR to be inferior to pathological nodal staging in node-positive colon cancer patients (46, 47).

### Lymph node ratio in metastatic CRC

LNR has been also evaluated as a prognostic marker in patients with colorectal cancer and liver metastases.

High LNR was significantly associated with lower 3-year relapse free survival (RFS) in patients with liver-limited disease undergoing curative resection, as observed by Deng Y et al. (43).

Alexandrescu ST et al. evaluated the role of LNR in predicting prognosis of patients with synchronous liver metastases and found that LNR was the only independent predictor of both DFS and OS (44).

LNR has been correlated with burden of liver metastases as well, as shown by Ahmad A et al. in their analysis of 53 stage IV colorectal cancer patients (45); authors found that high LNR status predicted the presence of more than 3 liver lesions and poorer OS.

### Log odds of positive lymph nodes

Log Odds of Positive Lymph Nodes (LODDS) is defined as the logarithm of the ratio of metastatic lymph nodes to negative lymph node (**Figure 1**).

The LODDS classification system has been tested with success in both breast and gastric cancer (56, 57). When applied to colon cancer, LODDS was proven effective in discriminating between patients with overlapping LNR values as shown by Wang J in a work already reported in this review (19).

Fang HY et al. compared the prognostic assessment of pathological nodal stage, LNR and LODDS using data collected retrospectively from 192 patients with resected colorectal cancer (48). All three variables correlated significantly with survival, yet LODDS was superior to the other categories in the multivariate analysis. Li T et al. confirmed the prognostic and clinic-pathological value of LODDS in a cohort of 389 patients with colorectal cancer undergoing curative surgery (49).

An interesting work by Occhionorelli et al. proved that LODDS was the only nodal category able to independently predict prognosis in 320 patients with colon cancer receiving emergency surgery (50).

LODDS was a reliable prognosticator in locally advanced rectal cancer as well, as reported in works by Lee CW et al. and Xu T et al. (51, 52). In particular, the latter work highlighted once more the importance of different staging approaches in improving the definition of prognosis in patients with lower LN yield.

LODDS has been proposed to complement existing staging classification, too. Pei JP et al. tried combining tumor stage with LODDS to classify 45,558 patients from the SEER database and found that the novel TLODDS classification has better discriminatory ability than current TNM staging (37).

However, criticism has emerged regarding simplicity of application of LODDS.

Baqar et al. compared LNR and LODDS in a cohort of 862 patients and found no difference in the prognostic impact of the two categories, suggesting LNR use is preferrable due to its ease of calculation (53). Song YX et al. analyzed data of 1,297 patients with colorectal cancer and found the LNR classification was superior to LODDS in assessing patient prognosis (54).

Summarizing, LNR is an independent and more accurate prognostic method for early colon cancer patients than AJCC TNM categories, even though no consensus has been reached on minimum number of lymph nodes to examine and on the cut-off to implement in existing staging systems. It can also be informative in the metastatic setting, since it has shown correlation to burden of liver metastases and survival in patients undergoing curative resection. It is a matter of discussion whether LODDS adds additional information to LNR and N staging.

In conclusion, both LNR and LODDS have been thoroughly evaluated as prognostic markers and should be evaluated for incorporation in upcoming staging classifications.

## Tumor deposits

Tumor deposits (TDs) are defined as discrete nodules of tumor cells in the bowel surrounding fat, lacking associated lymph node tissue and vascular or neural structures, which are found in 20-25% of colon cancer patients (**Figure 1**) (58).

Since its inclusion in the AJCC TNM staging system, TDs definition has changed considerably and, with every new edition, there has been an upstaging for patients with TDs between in up to 64% of cases (59). They were first defined as a separate entity in the 7^th^ edition of the TNM classification, with the introduction of the pN1c category, categorizing the presence of TDs in the absence of LNMs, whereas, in presence of lymph node metastases, TD status is discarded.

However, presence of TDs seems to be prognostically of equal importance to N status and its evaluation should not be restricted to cases in which pathological lymph nodes are absent (60).

In fact, a retrospective analysis performed by Shen F on 19,991 patients with colorectal cancer pooled from the SEER database found that the N1c category is associated with a prognosis similar to that of the N1b category (61). Mayo et al. performed a different analysis on the same database and showed that presence of TDs is associated with lower 3-year OS in multivariable models (62). Interestingly, presence of TDs is associated with worsening hazard ratio in lower N stages. A phase III trial in colon cancer patients receiving adjuvant chemotherapy (IDEA France) also demonstrated a significantly higher risk of recurrence or death in patients with TDs, regardless of LNM substatus (63).

Moreover, factoring of TDs should not be dichotomic. TDs should rather be considered as a quantitative variable, with a higher number of TDs predicting worse survival (58).

A retrospective analysis performed by Pricolo EV et al. in stage III colon cancer patients showed how patients included in pN1c staging category with ≥ 3 TDs had a worse overall survival than those with < 3 TDs, with a prognosis resembling that of pN2 patients (64). Zheng K et al. identified a cutoff of 4 or more TDs to predict poorer disease specific survival using data pooled from SEER database (65).

A similar conclusion was produced by Wang S et al. using data from 39,155 colorectal cancer patients within the SEER database (66). Authors found that the prognostic value of one TD is equivalent to that of two metastatic LNs based on the comparison of cause-specific survival rates and proposed this approach to be superior to the N1c staging in stratifying patient prognosis.

Research produced by Mirkin KA et al. and Zheng P et al. in patients with stage III colon cancer pooled from the National Cancer Data Base and SEER registry found that the presence of both TDs and LN metastases confers additive risk. Presence of both elements was, in fact, associated with significantly worse survival than each of these risk factors alone (67, 68).

A recent *post hoc* analysis of the CALGB/SWOG 80702 phase III study by Cohen R et al. suggested that combining the number of TDs to that of pathological lymph nodes improves the prognostic accuracy of current TNM staging (69). Combining TD and the number of lymph node metastases, 104 of the 1470 patients included in the analysis were re-staged as pN2 and showed significantly worse outcomes than those patients confirmed as pN1.

Other features of TDs have been investigated beyond their number.

A retrospective review classified TDs in invasive-type TD (iTD) (vascular invasion, lymphatic invasion, perineural invasion and undefined cancer clusters) or nodular-type TD (nTD) (cancer aggregates without iTD component): DFS was significantly shorter in both node-negative and node-positive, iTD/nTD+ patients compared to TD- patients. Among node-negative patients, disease-specific survival (DSS) differed significantly between the iTD/nTD+ and TD− groups, while in node-positive patients presence of nTD had no impact on DSS (70).

A more accurate staging of these patients may also help to improve adjuvant treatment strategies. Currently, in fact, patients with TDs but no metastatic LNs are less likely to receive adjuvant chemotherapy (52% vs 74%) and have longer delay to treatment initiation, as shown by Wong-Chong N et al. These patients are also reported to be younger and to have more adverse tumor features (71).

Adequate selection for adjuvant treatment is even more relevant, considering that the number of TDs reported does not impact the benefit of adjuvant chemotherapy (72).

Finally, TDs have been evaluated as prognostic indicator in metastatic CRC as well. Lin Q et al. evaluated 146 patients with synchronous colorectal liver metastases undergoing simultaneous resection of primary tumor and metastatic lesions. Authors found that the presence of TDs was associated with significantly shorter DFS, regardless of LN status (73).

All the evidence reviewed is summarized in **Table 2**.

**Table 2:** Evidence regarding TDs implementation.

Authors	Subject	Major Findings	Reference
Nagtegaal ID et al.	Staging TDs	Presence of TDs is at least of equal importance to N status and its factoring should not be restricted to cases in which LN are absent.	(59, 60)
Shen F et al.	Staging TDs	Cancer specific survival difference between N1b and N1c is not statistically significant	(61)
Mayo et al.	Presence of TDs	Presence of TDs predicts poorer survival, especially in lower N stages	(62)
Pricolo VE et al.; Brouwer NPM et al; Zheng K et al	Number of TDs	TDs number is associated with worse survival	(58, 64, 65)
Mirkin KA et al.; Zheng P et al.	Presence of TDs and LNMs	Presence of both TDs and LNM was associated with worse survival than with each factor alone	(67, 68)
Nagtegaal ID et al.; Wang S et al.; Cohen R et al.;	Number of TDs	TDs should be added to final N count. According to Wang S et al. one TD should be considered as two LNMs	(59, 60, 66, 69)
Yamano T et al.	TDs subclassification	Classifying TDs in invasive-type and nodular-type TDs may improve prognostic value	(70)
Lin et al.	TDs evaluation in metastatic disease	Presence of TDs is associated with worse survival in patients undergoing simultaneous resection for liver colorectal metastases	(73)

LNM, Lymph Node Metastasis; TD, Tumor Deposit.

Summarizing, presence of TDs is at least of equal importance to N status and its factoring should not be restricted to cases in which lymph node metastases are absent, considering also that both features confer additive risk. Factoring of TDs should not be dichotomic as higher count of deposits predicts poorer survival. Presence of TDs is also informative in metastatic CRC, as it is associated with worse survival in patients undergoing simultaneous resection for liver colorectal metastases.

In conclusion, TDs can influence patient prognosis significantly and should be highly considered when evaluating patient prognosis and indications to adjuvant treatment.

## Extranodal extension

Extranodal extension (ENE) is defined as the extension of tumor cells through the nodal capsule into the perinodal fatty tissue (**Figure 1**). Current AJCC TNM classification in colorectal cancer does not account for presence of ENE, even though it could theoretically identify a more aggressive disease (74).

Evidence has accumulated regarding its prognostic significance in several malignancies, including breast, head and neck, gastro-esophageal, prostate and bladder cancer (75–79).

Early evidence of its role in colorectal cancer was collected in a systematic review of literature by Wind J et al. which included 4 series of patients with lower gastrointestinal tract malignancies, where presence of ENE identified patients with significantly worse long-term prognosis (80).

More recently Veronese N et al. published a new systematic review with meta-analysis evaluating 1,336 patients with colorectal cancer from 13 different trials (81). Authors reported ENE was associated significantly with higher stage and grade of disease, increased risk of all-cause mortality (HR = 1.69, 95% CI 1.32–2.17, P < 0.0001) and increased risk of recurring disease (HR = 2.31, 95% CI 1.54–3.44, P < 0.0001).

Further evidence has since emerged. Ambe PC et al. reported data from a cohort of 147 patient with node-positive colorectal cancer, suggesting extranodal extension predicts higher risk of cancer-related death (OR= 0.44, p = 0.021) and shorter median OS (30.5 ± 42 months vs. 51.0 ± 33, p = 0.02).

Kim CW et al. reported the results of a single-institution analysis of 2,346 patients with colorectal cancer receiving curative surgery (6). Authors found that ENE was associated with younger age, more advanced tumor stage, presence of both lymphovascular invasion (LVI) and perineural invasion (PNI) in both colon and rectal cancer. Interestingly, ENE frequency was described to be increasing from the right colon to the left colon and the presence of this pathological feature was reported to independently predict DFS regardless of tumor location in patients who do not receive adjuvant chemotherapy.

A paper produced by Li T et al., already mentioned in our work in relation to LODDS, confirmed these findings in a different cohort (49). ENE frequency was once more reported to increase in distal tumors and its presence was associated with worse prognosis in both colon and rectal cancer patients.

Summarizing, presence of ENE is associated with increased risk of recurrence and worse survival. It is also frequently associated with other “high-risk” features such as higher tumor grade and stage.

All the evidence available is therefore in favor of ENE implementation in current classifications to improve patient stratification and selection for treatment intensification.

## Conclusion

Current management of early colorectal cancer is based on the existing 8^th^ edition of the TNM classification. However, emerging pathological features as described in this review, can significantly modify the prognosis of patients within a same stage group. It should be noted that most of the evidence reviewed in this paper is based on retrospective analysis, which carry intrinsic limitations. Nevertheless, until prospective evidence is available to support implementation of LNR, TDs and ENE in clinical practice, clinicians should evaluate these features in addition to traditional staging system on a patient basis in order to guide treatment and follow up in cases where risk assessment is not straight-forward.

In the era of precision medicine and amidst attempts to de-escalate intensity of adjuvant treatments, it appears fundamental to adequately select patients with worse prognosis who require a more aggressive management.

We believe that both pathologist and clinicians should factor LNR, LODDS, TD and ENE when assessing a patient’s outcome and when selecting individuals for a more intensive treatment and follow-up strategy.

## Author contributions

GA contributed to conception and design of the review. GA, MP, BF, and SF wrote the first draft of the manuscript. GA, MP, BF, and SF wrote sections of the manuscript. All authors contributed to manuscript revision, read, and approved the submitted version

## Conflict of interest

The authors declare that the research was conducted in the absence of any commercial or financial relationships that could be construed as a potential conflict of interest.

## Publisher’s note

All claims expressed in this article are solely those of the authors and do not necessarily represent those of their affiliated organizations, or those of the publisher, the editors and the reviewers. Any product that may be evaluated in this article, or claim that may be made by its manufacturer, is not guaranteed or endorsed by the publisher.

## References

[1] Sung H Ferlay J Siegel RL Laversanne M Soerjomataram I Jemal A. Global cancer statistics 2020: globocan estimates of incidence and mortality worldwide for 36 cancers in 185 countries. CA Cancer J Clin (2021) 71(3):209–49. doi: 10.3322/caac.21660 33538338

[2] Siegel RL Miller KD Fedewa SA Ahnen DJ Meester RGS Barzi A. Colorectal cancer statistics, 2017. CA Cancer J Clin (2017) 67(3):177–93. doi: 10.3322/caac.21395 28248415

[3] Siegel RL Miller KD Fuchs HE Jemal A. Cancer statistics, 2021. CA Cancer J Clin (2021) 71(1):7–33. doi: 10.3322/caac.21654 33433946

[4] Brierley JD Gospodarowicz MK Wittekind C UICC. TNM classification of malignant tumours, 8 th edition due december 2016. Union Int Cancer Control (2017) 1:73–76.

[5] Weiser MR. AJCC 8th edition: colorectal cancer. Ann Surg Oncol United States (2018) 25:1454–5. doi: 10.1245/s10434-018-6462-1 29616422

[6] Kim CW Kim J Park Y Cho DH Lee JL Yoon YS. Prognostic implications of extranodal extension in relation to colorectal cancer location. Cancer Res Treat (2019) 51(3):1135–43. doi: 10.4143/crt.2018.392 PMC6639205 30514068

[7] Le Voyer TE Sigurdson ER Hanlon AL Mayer RJ Macdonald JS Catalano PJ. Colon cancer survival is associated with increasing number of lymph nodes analyzed: a secondary survey of intergroup trial INT-0089. J Clin Oncol (2003) 21(15):2912–9. doi: 10.1200/JCO.2003.05.062 12885809

[8] Roth AD Delorenzi M Tejpar S Yan P Klingbiel D Fiocca R. Integrated analysis of molecular and clinical prognostic factors in stage II/III colon cancer. J Natl Cancer Inst (2012) 104(21):1635–46. doi: 10.1093/jnci/djs427 23104212

[9] Aparicio T. Tumor microsatellite-instability status as a predictor of benefit from fluorouracil-based adjuvant chemotherapy for colon cancer. Colon Rectum (2013) 7(1):46–8. doi: 10.1007/s11725-013-0437-y PMC3584639 12867608

[10] Jin M-L Gong Y Pei Y-C Ji P Hu X Shao Z-M. Modified lymph node ratio improves the prognostic predictive ability for breast cancer patients compared with other lymph node staging systems. Breast (2020) 49:93–100. doi: 10.1016/j.breast.2019.11.003 31783315 PMC7375622

[11] Zhu J Xue Z Zhang S Guo X Zhai L Shang S. Integrated analysis of the prognostic role of the lymph node ratio in node-positive gastric cancer: A meta-analysis. Int J Surg (2018) :57:76–83. doi: 10.1016/j.ijsu.2018.08.002 30103072

[12] Yukawa N Aoyama T Tamagawa H Tamagawa A Atsumi Y Kawahara S. The lymph node ratio is an independent prognostic factor in esophageal cancer patients who receive curative surgery. In Vivo (2020) 34(4):2087–93. doi: 10.21873/invivo.12012 PMC7439888 32606187

[13] Mansour J Sagiv D Alon E Talmi Y. Prognostic value of lymph node ratio in metastatic papillary thyroid carcinoma. J Laryngol Otol (2018) 132(1):8–13. doi: 10.1017/S0022215117002250 29122022

[14] Kim J Park J Park H Choi MS Jang HW Kim TH. Metastatic lymph node ratio for predicting recurrence in medullary thyroid cancer. Cancers (2021) 13:5842. doi: 10.3390/cancers13225842 34830996 PMC8616059

[15] Zhou J Lin Z Lyu M Chen N Liao H Wang Z. Prognostic value of lymph node ratio in non-small-cell lung cancer: a meta-analysis. Jpn J Clin Oncol (2020) 50(1):44–57. doi: 10.1093/jjco/hyz120 31735973

[16] Bu DD Ferrandino R Robinson EM Liu S Miles BA Teng MS. Lymph node ratio in HPV-associated oropharyngeal cancer: identification of a prognostic threshold. Laryngoscope (2021) 131(1):E184–9. doi: 10.1002/lary.28689 32348558

[17] Tong GJ Zhang GY Liu J Zheng ZZ Chen Y Niu PP. Comparison of the eighth version of the American joint committee on cancer manual to the seventh version for colorectal cancer: A retrospective review of our data. World J Clin Oncol (2018) 9(7):148–61. doi: 10.5306/wjco.v9.i7.148 PMC6230917 30425940

[18] Gao P Song Y Wang Z Xu Y Tong L Sun J. Is the prediction of prognosis not improved by the seventh edition of the TNM classification for colorectal cancer? analysis of the surveillance, epidemiology, and end results (SEER) database. BMC Cancer (2013) 13:123. doi: 10.1186/1471-2407-13-123 23496812 PMC3651725

[19] Wang J Hassett JM Dayton MT Kulaylat MN. Lymph node ratio: role in the staging of node-positive colon cancer. Ann Surg Oncol (2008) 15(6):1600–8. doi: 10.1245/s10434-007-9716-x 18327530

[20] Chin CC Wang JY Yeh CY Kuo YH Huang WS Yeh CH. Metastatic lymph node ratio is a more precise predictor of prognosis than number of lymph node metastases in stage III colon cancer. Int J Colorectal Dis (2009) 24(11):1297–302. doi: 10.1007/s00384-009-0738-7 19479270

[21] Ceelen W Van Nieuwenhove Y Pattyn P. Prognostic value of the lymph node ratio in stage III colorectal cancer: a systematic review. Ann Surg Oncol (2010) 17(11):2847–55. doi: 10.1245/s10434-010-1158-1 20559741

[22] Berger AC Sigurdson ER LeVoyer T Hanlon A Mayer RJ Macdonald JS. Colon cancer survival is associated with decreasing ratio of metastatic to examined lymph nodes. J Clin Oncol (2005) 23(34):8706–12. doi: 10.1200/JCO.2005.02.8852 16314630

[23] Li Destri G Barchitta M Pesce A Latteri S Bosco D Di Cataldo A. Predictive value of the number of harvested lymph nodes and cut-off for lymph node ratio in the prognosis of stage ii and iii colorectal cancer patients. J Invest Surg (2019) 32(1):1–7. doi: 10.1080/08941939.2017.1369605 28972442

[24] Shimomura M Ikeda S Takakura Y Kawaguchi Y Tokunaga M Egi H. Adequate lymph node examination is essential to ensure the prognostic value of the lymph node ratio in patients with stage III colorectal cancer. Surg Today (2011) 41(10):1370–9. doi: 10.1007/s00595-010-4446-2 21922359

[25] In JP Choi GS Soo HJ. Nodal stage of stage III colon cancer: the impact of metastatic lymph node ratio. J Surg Oncol (2009) 100(3):240–3. doi: 10.1002/jso.21273 19330780

[26] Rosenberg R Friederichs J Schuster T Gertler R Maak M Becker K. Prognosis of patients with colorectal cancer is associated with lymph node ratio: a single-center analysis of 3,026 patients over a 25-year time period. Ann Surg (2008) 248(6):968–78. doi: 10.1097/SLA.0b013e318190eddc 19092341

[27] Peschaud F Benoist S Julié C Beauchet A Penna C Rougier P. The ratio of metastatic to examined lymph nodes is a powerful independent prognostic factor in rectal cancer. Ann Surg (2008) 248(6):1067–73. doi: 10.1097/SLA.0b013e31818842ec 19092352

[28] MacEdo F Sequeira H Ladeira K Bonito N Viana C Martins S. Metastatic lymph node ratio as a better prognostic tool than the TNM system in colorectal cancer. Future Oncol (2021) 17(12):1519–32. doi: 10.2217/fon-2020-0993 33626938

[29] Parnaby CN Scott NW Ramsay G Mackay C Samuel L Murray GI. Prognostic value of lymph node ratio and extramural vascular invasion on survival for patients undergoing curative colon cancer resection. Br J Cancer (2015) 113(2):212–9. doi: 10.1038/bjc.2015.211 PMC4506392 26079302

[30] Isik A Peker K Firat D Yilmaz B Sayar I Idiz O. Importance of metastatic lymph node ratio in non-metastatic, lymph node-invaded colon cancer: A clinical trial. Med Sci Monit. (2014) 20:1369–75. doi: 10.12659/MSM.890804 PMC4136934 25087904

[31] Sabbagh C Mauvais F Cosse C Rebibo L Joly JP Dromer D. A lymph node ratio of 10% is predictive of survival in stage III colon cancer: A french regional study. Int Surg (2014) 99(4):344–53. doi: 10.9738/INTSURG-D-13-00052 PMC4114359 25058763

[32] Shinto E Ike H Hida J-I Kobayashi H Hashiguchi Y Kajiwara Y. Marked impact of tumor location on the appropriate cutoff values and the prognostic significance of the lymph node ratio in stage III colon cancer: a multi-institutional retrospective analysis. J Gastroenterol (2019) 54(7):597–607. doi: 10.1007/s00535-018-01539-5 30607613

[33] Zhang C-H Li Y-Y Zhang Q-W Biondi A Fico V Persiani R. The prognostic impact of the metastatic lymph nodes ratio in colorectal cancer. Front Oncol (2018) 8. doi: 10.3389/fonc.2018.00628 PMC6305371 30619762

[34] Sugimoto K Sakamoto K Tomiki Y Goto M Kotake K Sugihara K. Proposal of new classification for stage III colon cancer based on the lymph node ratio: analysis of 4,172 patients from multi-institutional database in Japan. Ann Surg Oncol (2015) 22(2):528–34. doi: 10.1245/s10434-014-4015-9 25160735

[35] Wang LP Wang HY Cao R Zhu C Wu XZ. Proposal of a new classification for stage III colorectal cancer based on the number and ratio of metastatic lymph nodes. World J Surg (2013) 37(5):1094–102. doi: 10.1007/s00268-013-1940-x 23385643

[36] Pei J-P Zhang R Zhang N-N Zeng Y-J Sun Z Ma S-P. Screening and validation of a novel T stage-lymph node ratio classification for operable colon cancer. Ann Transl Med (2021) 9(20):1513. doi: 10.21037/atm-21-3170 34790719 PMC8576719

[37] Pei J-P Zhao Z-M Sun Z Gu W-J Zhu J Zhu J. Development and validation of a novel classification scheme for combining pathological T stage and log odds of positive lymph nodes for colon cancer. Eur J Surg Oncol (2022) 48(1):228–36. doi: 10.1016/j.ejso.2021.09.005 34531116

[38] Madbouly KM Abbas KS Hussein AM. Metastatic lymph node ratio in stage III rectal carcinoma is a valuable prognostic factor even with less than 12 lymph nodes retrieved: a prospective study. Am J Surg (2014) 207(6):824–31. doi: 10.1016/j.amjsurg.2013.07.022 24112666

[39] Chen L Huang X Song Z. The value of lymph node ratio in the prediction of rectal cancer patient survival after preoperative chemoradiotherapy. Int J Clin Exp Pathol (2018) 11(12):5992–6001. 31949687 PMC6963083

[40] Lykke J Jess P Roikjaer O. The prognostic value of lymph node ratio in a national cohort of rectal cancer patients. Eur J Surg Oncol J Eur Soc Surg Oncol Br Assoc Surg Oncol (2016) 42(4):504–12. doi: 10.1016/j.ejso.2016.01.012 26856955

[41] Junginger T Goenner U Lollert A Hollemann D Berres M Blettner M. The prognostic value of lymph node ratio and updated TNM classification in rectal cancer patients with adequate versus inadequate lymph node dissection. Tech Coloproctol (2014) 18(9):805–11. doi: 10.1007/s10151-014-1136-x 24643761

[42] Karjol U Jonnada P Chandranath A Cherukuru S. Lymph node ratio as a prognostic marker in rectal cancer survival: A systematic review and meta-analysis. Cureus (2020) 12(5):e8047. doi: 10.7759/cureus.8047 32399378 PMC7216312

[43] Deng Y Peng J Zhao Y Sui Q Zhao R Lu Z. Lymph node ratio as a valuable prognostic factor for patients with colorectal liver-only metastasis undergoing curative resection. Cancer Manag Res (2018) 10:2083–94. doi: 10.2147/CMAR.S169029 PMC6054757 30140159

[44] Alexandrescu ST Selaru FM Diaconescu AS Zlate CA Blanita D Grigorie RT. Prognostic value of lymph node ratio in patients with resected synchronous colorectal liver metastases and less than 12 examined lymph nodes. J Gastrointest Surg Off J Soc Surg Aliment Tract (2022) 26(1):141–9. doi: 10.1007/s11605-021-05079-x 34258674

[45] Ahmad A Reha J Saied A Espat NJ Somasundar P Katz SC. Association of primary tumor lymph node ratio with burden of liver metastases and survival in stage IV colorectal cancer. Hepatobiliary Surg Nutr (2017) 6(3):154–61. doi: 10.21037/hbsn.2016.08.08 PMC5474451 28652998

[46] Jakob MO Guller U Ochsner A Oertli D Zuber M Viehl CT. Lymph node ratio is inferior to pN-stage in predicting outcome in colon cancer patients with high numbers of analyzed lymph nodes. BMC Surg (2018) 18(1):81. doi: 10.1186/s12893-018-0417-0 30285691 PMC6171184

[47] Mohan HM Walsh C Kennelly R Ng CH O’Connell PR Hyland JM. The lymph node ratio does not provide additional prognostic information compared with the N1/N2 classification in stage III colon cancer. Color Dis Off J Assoc Coloproctology Gt Britain Irel (2017) 19(2):165–71. doi: 10.1111/codi.13410 27317165

[48] Fang HY Yang H He ZS Zhao H Fu ZM Zhou FX. Log odds of positive lymph nodes is superior to the number- and ratio-based lymph node classification systems for colorectal cancer patients undergoing curative (R0) resection. Mol Clin Oncol (2017) 6(5):782–8. doi: 10.3892/mco.2017.1203 PMC5431519 28529752

[49] Li T Yang Y Wu W Fu Z Cheng F Qiu J. Prognostic implications of ENE and LODDS in relation to lymph node-positive colorectal cancer location. Transl Oncol (2021) 14(11):101190. doi: 10.1016/j.tranon.2021.101190 34403906 PMC8367836

[50] Occhionorelli S Andreotti D Vallese P Morganti L Lacavalla D Forini E. Evaluation on prognostic efficacy of lymph nodes ratio (LNR) and log odds of positive lymph nodes (LODDS) in complicated colon cancer: the first study in emergency surgery. World J Surg Oncol (2018) 16(1):186. doi: 10.1186/s12957-018-1483-6 30213260 PMC6137917

[51] Xu T Zhang L Yu L Zhu Y Fang H Chen B. Log odds of positive lymph nodes is an excellent prognostic factor for patients with rectal cancer after neoadjuvant chemoradiotherapy. Ann Transl Med (2021) 9(8):637. doi: 10.21037/atm-20-7590 33987335 PMC8106017

[52] Lee CW Wilkinson KH Sheka AC Leverson GE Kennedy GD. The log odds of positive lymph nodes stratifies and predicts survival of high-risk individuals among stage III rectal cancer patients. Oncologist (2016) 21(4):425–32. doi: 10.1634/theoncologist.2015-0441 PMC4828122 26975865

[53] Baqar AR Wilkins S Wang W Oliva K McMurrick P. Log odds of positive lymph nodes is prognostically equivalent to lymph node ratio in non-metastatic colon cancer. BMC Cancer (2020) 20(1):762. doi: 10.1186/s12885-020-07260-y 32795292 PMC7427861

[54] Song YX Gao P Wang ZN Tong LL Xu YY Sun Z. Which is the most suitable classification for colorectal cancer, log odds, the number or the ratio of positive lymph nodes? PloS One (2011) 6(12):e28937. doi: 10.1371/journal.pone.0028937 22174929 PMC3236772

[55] Lv Y Feng QY Bin LS YH M Xu YQ Zheng P. Exploration of exact significance of lymph node ratio and construction of a novel stage in colon cancer with no distant metastasis. Cancer Manag Res (2019) 11:6841–54. doi: 10.2147/CMAR.S203533 PMC6664259 31440082

[56] Sun Z Xu Y Li DM Wang ZN Zhu GL Huang BJ. Log odds of positive lymph nodes: A novel prognostic indicator superior to the number-based and the ratio-based n category for gastric cancer patients with R0 resection. Cancer (2010) 116(11):2571–80. doi: 10.1002/cncr.24989 20336791

[57] Vinh-Hung V Verschraegen C Promish DI Cserni G Van de Steene J Tai P. Ratios of involved nodes in early breast cancer. Breast Cancer Res (2004) 6(6). doi: 10.1186/bcr934 PMC1064081 15535850

[58] Brouwer NPM Nagtegaal ID. Tumor deposits improve staging in colon cancer: what are the next steps? Ann Oncol (2021) 32(10):1209–11. doi: 10.1016/j.annonc.2021.08.1751 34416364

[59] Nagtegaal ID Tot T Jayne DG McShane P Nihlberg A Marshall HC. Lymph nodes, tumor deposits, and TNM: are we getting better? J Clin Oncol Off J Am Soc Clin Oncol (2011) 29(18):2487–92. doi: 10.1200/JCO.2011.34.6429 21555695

[60] Nagtegaal ID Knijn N Hugen N Marshall HC Sugihara K Tot T. Tumor deposits in colorectal cancer: improving the value of modern staging-a systematic review and meta-analysis. J Clin Oncol Off J Am Soc Clin Oncol (2017) 35(10):1119–27. doi: 10.1200/JCO.2016.68.9091 28029327

[61] Shen F Hong X. Prognostic value of N1c in colorectal cancer: a large population-based study using propensity score matching. Int J Colorectal Dis (2019) 34(8):1375–83. doi: 10.1007/s00384-019-03328-9 31201493

[62] Mayo E Llanos AAM Yi X Duan S-Z Zhang L. Prognostic value of tumour deposit and perineural invasion status in colorectal cancer patients: a SEER-based population study. Histopathology (2016) 69(2):230–8. doi: 10.1111/his.12936 26802566

[63] Delattre J-F Cohen R Henriques J Falcoz A Emile J-F Fratte S. Prognostic value of tumor deposits for disease-free survival in patients with stage III colon cancer: a *post hoc* analysis of the idea france phase III trial (PRODIGE-GERCOR). J Clin Oncol (2020) 38(15):1702–10. doi: 10.1200/JCO.19.01960 32167864

[64] Pricolo VE Steingrimsson J McDuffie TJ McHale JM McMillen B Shparber M. Tumor deposits in stage iii colon cancer: correlation with other histopathologic variables, prognostic value, and risk stratification-time to consider “N2c”. Am J Clin Oncol (2020) 43(2):133–8. doi: 10.1097/COC.0000000000000645 PMC7004443 31764018

[65] Zheng K Zheng N Xin C Zhou L Sun G Wen R. The prognostic significance of tumor deposit count for colorectal cancer patients after radical surgery. Gastroenterol Res Pract (2020) 2020:1848252. doi: 10.1155/2020/2052561 33014036 PMC7520671

[66] Wang S Guan X Ma M Zhuang M Ma T Liu Z. Reconsidering the prognostic significance of tumour deposit count in the TNM staging system for colorectal cancer. Sci Rep (2020) 10(1):1–8. doi: 10.1038/s41598-019-57041-2 31919408 PMC6952424

[67] Mirkin KA Kulaylat AS Hollenbeak CS Messaris E. Prognostic significance of tumor deposits in stage iii colon cancer. Ann Surg Oncol (2018) 25(11):3179–84. doi: 10.1245/s10434-018-6661-9 30083832

[68] Zheng P Chen Q Li J Jin C Kang L Chen D. Prognostic significance of tumor deposits in patients with stage III colon cancer: a nomogram study. J Surg Res (2020) 245:475–82. doi: 10.1016/j.jss.2019.07.099 31446189

[69] Cohen R Shi Q Meyers J Jin Z Svrcek M Fuchs C. Combining tumor deposits with the number of lymph node metastases to improve the prognostic accuracy in stage III colon cancer: a *post hoc* analysis of the CALGB/SWOG 80702 phase III study (Alliance)(☆). Ann Oncol Off J Eur Soc Med Oncol (2021) 32(10):1267–75. doi: 10.1016/j.annonc.2021.07.009 PMC8719434 34293461

[70] Yamano T Semba S Noda M Yoshimura M Kobayashi M Hamanaka M. Prognostic significance of classified extramural tumor deposits and extracapsular lymph node invasion in T3-4 colorectal cancer: A retrospective single-center study. BMC Cancer (2015) 15(1):1–9. doi: 10.1186/s12885-015-1885-6 26545360 PMC4635537

[71] Wong-Chong N Motl J Hwang G Nassif GJJ Albert MR Monson JRT. Impact of tumor deposits on oncologic outcomes in stage III colon cancer. Dis Colon Rectum (2018) 61(9):1043–52. doi: 10.1097/DCR.0000000000001152 30086053

[72] Shi M Zhang H Yao G Wu J Zhu C Zhang X. The role of tumor deposits in predicting the efficacy of chemotherapy in stage III colon cancer. Front Oncol (2020) 10. doi: 10.3389/fonc.2020.586603 PMC7591764 33154948

[73] Lin Q Wei Y Ren L Zhong Y Qin C Zheng P. Tumor deposit is a poor prognostic indicator in patients who underwent simultaneous resection for synchronous colorectal liver metastases. Onco Targets Ther (2015) 8:233–40. doi: 10.2147/OTT.S71414 PMC4309783 25653544

[74] Stitzenberg KB Meyer AA Stern SL Cance WG Calvo BF Klauber-DeMore N. Extracapsular extension of the sentinel lymph node metastasis: a predictor of nonsentinel node tumor burden. Ann Surg (2003) 237(5):603–7. doi: 10.1097/01.SLA.0000064361.12265.9A PMC1514520 12724626

[75] Huang SH Chernock R O’Sullivan B Fakhry C. Assessment criteria and clinical implications of extranodal extension in head and neck cancer. Am Soc Clin Oncol Educ Book Am Soc Clin Oncol Annu Meet (2021) 41(41):265–78. doi: 10.1200/EDBK_320939 34010048

[76] Ma XX Yang X Yang W Shui R. Prognostic value of extranodal extension in axillary lymph node-positive breast cancer. Sci Rep (2021) 11(1):1–12. doi: 10.1038/s41598-021-88716-4 33953240 PMC8099896

[77] Alakus H Hölscher AH Grass G Hartmann E Schulte C Drebber U. Extracapsular lymph node spread: a new prognostic factor in gastric cancer. Cancer (2010) 116(2):309–15. doi: 10.1002/cncr.24764 19950124

[78] Ahn TS Kim HS Jeong CW Kwak C Kim HH Ku JH. Extracapsular extension of pelvic lymph node metastasis is an independent prognostic factor in bladder cancer: a systematic review and meta-analysis. Ann Surg Oncol (2015) 22(11):3745–50. doi: 10.1245/s10434-014-4359-1 25613388

[79] Griebling TL Ozkutlu D See WA Cohen MB. Prognostic implications of extracapsular extension of lymph node metastases in prostate cancer. Mod Pathol an Off J United States Can Acad Pathol Inc (1997) 10(8):804–9. 9267823

[80] Wind J Lagarde SM Ten Kate FJW Ubbink DT Bemelman WA van Lanschot JJB. A systematic review on the significance of extracapsular lymph node involvement in gastrointestinal malignancies. Eur J Surg Oncol J Eur Soc Surg Oncol Br Assoc Surg Oncol (2007) 33(4):401–8. doi: 10.1016/j.ejso.2006.11.001 17175130

[81] Veronese N Nottegar A Pea A Solmi M Stubbs B Capelli P. Prognostic impact and implications of extracapsular lymph node involvement in colorectal cancer: a systematic review with meta-analysis. Ann Oncol (2016) 27(1):42–8. doi: 10.1093/annonc/mdv494 26483050

